# Epigenetic Factors in Cancer Risk: Effect of Chemical Carcinogens on Global DNA Methylation Pattern in Human TK6 Cells

**DOI:** 10.1371/journal.pone.0034674

**Published:** 2012-04-11

**Authors:** Ali M. Tabish, Katrien Poels, Peter Hoet, Lode Godderis

**Affiliations:** 1 Department of Occupational, Environmental and Insurance Medicine, Katholieke Universiteit Leuven, Leuven, Belgium and; 2 IDEWE, External Service for Prevention and Protection at Work, Heverlee, Belgium; Università di Napoli Federico II, Italy

## Abstract

In the current study, we assessed the global DNA methylation changes in human lymphoblastoid (TK6) cells *in vitro* in response to 5 direct and 10 indirect-acting genotoxic agents. TK6 cells were exposed to the selected agents for 24 h in the presence and/or absence of S9 metabolic mix. Liquid chromatography-mass spectrometry was used for quantitative profiling of 5-methyl-2′-deoxycytidine. The effect of exposure on 5-methyl-2′-deoxycytidine between control and exposed cultures was assessed by applying the marginal model with correlated residuals on % global DNA methylation data. We reported the induction of global DNA hypomethylation in TK6 cells in response to S9 metabolic mix, under the current experimental settings. Benzene, hydroquinone, styrene, carbon tetrachloride and trichloroethylene induced global DNA hypomethylation in TK6 cells. Furthermore, we showed that dose did not have an effect on global DNA methylation in TK6 cells. In conclusion we report changes in global DNA methylation as an early event in response to agents traditionally considered as genotoxic.

## Introduction

Environmental carcinogens are a known risk factor of human cancer [1]. In its classical model, carcinogenesis initiates and proceeds through changes in the genome (i.e., genetic effects) [2]. Thus, measuring carcinogen-induced DNA damage i.e., DNA adducts formation and cross-linking, and DNA mutations have been employed in classic cancer risk assessment approaches, e.g., Ames test, comet assay and micronucleus assay [3]–[5]. Carcinogen-induced DNA damage is an important early event during the initiation phase of carcinogenesis, which reflects a permanent and irreversible change in the initiated cells [6], [7]. However, initiation *per se* in a classical carcinogenesis model is not sufficient for tumor development, which results from broader alterations in the cellular homeostasis, mainly because of the inability of initiated cells to properly control and regulate the gene expression [8].

Exposure to genotoxic carcinogens, in addition to their genetic effects, might involve a variety of non-genotoxic effects in cells [9]. Non-genotoxic effects in cells may play an important role in cancer development [10]. Evidence suggest that non-genotoxic alterations in cells, e.g., alterations in cellular epigenome, could result in the emergence of epigenetically reprogrammed cells [11]. These epigenetically reprogrammed cells show an epigenetic profile similar to that frequently observed in cancer cells, such as altered histone modification patterns, hypomethylation of DNA repetitive elements and proto-oncogenes and hypermethylation of tumor suppressor genes. Altered epigenetic status confers genome instability and loss of controlled growth signals, typically observed in cancer cells [12]. Epigenetic alterations rather than specific genetic mutations *per se* are reported for the clonal expansion of altered hepatic preneoplastic foci and tumor development [13].

Recently, a number of studies reported that the carcinogenic effects induced by 2-acetylaminofluorene, tamoxifen, trichloroethylene, aflatoxin B1, ochratoxin, nickel and chromium do not follow a classical carcinogenesis model, but rather involve a spectrum of cellular alterations encompassing the epigenetics. [8], [14]–[16]. Epigenetic factors play an important role in cancer etiology; however, there is insufficient knowledge in linking epigenetic factors to environmental carcinogenesis in premalignant tissue [17]. Based on increasingly documented epigenetic changes in cancer etiology, the goal of this study is to assess if alterations in global DNA methylation are an early cellular event in response to genotoxic carcinogens with a well-known mode of action (adducts forming and cross-linking agents). In this study, we used 5 direct and 10 indirect- acting genotoxic carcinogens to expose human lymphoblastoid cells (TK6) for 24 h. TK6 cells were exposed to carcinogens at 3 dose levels (low, medium and high) in duplicates. S9 metabolic mix was added in cultures in half of the experiments because indirect- acting carcinogens require S9 metabolic mix to become functional carcinogens. We used human thymidine kinase heterozygote TK6 cells in this study because they express wild-type p53, grow rapidly in suspension (population doubling time of 12–14 h), and are routinely used in genetic toxicology studies. After exposure, cells were harvested, DNA was extracted, hydrolyzed, and global DNA methylation levels were quantified in TK6 cells.

## Materials and Methods

### Cell Culture

TK6 cells were purchased from the European Collection of Cell Cultures (ECACC, Wiltshire, UK). Cells were divided into 15 treatment groups and 2 control groups (control S9−, control S9+), and cultured in RPMI 1640 medium containing 10% heat-inactivated horse serum, 100 U/ml penicillin, 100 µg/ml streptomycin and 2 mM l-glutamine at 5% CO2 and 37°C. Cells were maintained at a density of 10^6^ cells/ml and exposed for 24 h to carcinogens. We set up two biological replicates per chemical dose, 10 control S9− replicates, and 5 control S9+ replicates.

Due to the requirement of enzymatic biotransformation of procarcinogens to become active carcinogens, a mixture of S9 (1% v/v) from human liver was added to the culture in half of the experiments [18], [19]. Liver S9 fractions were obtained from Celsis (Neuss, Germany), and contained drug-metabolizing enzymes including the cytochromes P450, flavin monooxygenases, and UDP glucuronyl transferases. An exogenous NADPH-regenerating system (1.3 mM NADP+, 3.3 mM glucose-6-phosphate, 0.4 U/ml glucose-6-phosphate dehydrogenase, and 3.3 mM magnesium chloride; BD Biosciences, Erembodegem, Belgium) required by liver S9 for phase I oxidation was included in the experiments. Cells were exposed to carcinogen in duplicates with or without S9 metabolic mix.

### Chemicals, Viability Assays and Dose Selection

We selected chemicals with well-described genotoxic characteristics [20]. A list of the selected agents, their classification and exposure dose is given in Table S1. All chemicals were purchased from Sigma Aldrich, and dissolved and diluted in dimethylsulfoxide (DMSO). Viability assays were used to select doses per agent. We used 3-[4,5-dimethylthiazol-2-yl]-2,5-diphenyl tetrazolium bromide (MTT) viability assay [21], and also counted the proportions of living and dead cells using a Countess™ Automated Cell Counter (Invitrogen, Carlsbad, CA). Based on the viability assays, we selected three doses per chemical, i.e. a dose with 95% cellular viability (high dose), 1/10 of high dose (medium dose) and 1/100 of high dose (low dose).

### DNA Extraction, Concentration and Purity

After 24 h of treatment, cells were immediately processed for DNA extraction. DNA was extracted using Trizol® reagent with the PureLinkTM Micro-to-Midi System® according to the manufacturer's protocol (Invitrogen, Carlsbad, CA). DNA quantity and quality was measured by NanoDrop Spectrophotometry and Agilent 2100 bioanalyzer.

### Enzymatic Hydrolysis of DNA

Extracted DNA was hydrolyzed to individual deoxyribonucleosides in a simplified one-step procedure [22]. In short, DNA digest mix was prepared by adding 250 U Benzonase (Sigma Aldrich), 300 mU Phosphodiesterase I (Sigma Aldrich), and 200 U alkaline phosphatase (Sigma Aldrich) to 5 ml Tris-HCl buffer (pH 7.9, 20 mM) containing 100 mM NaCl and 20 mM MgCl_2_. 1 µg of extracted DNA from exposed and control samples was hydrolyzed in 100 µl of reaction by adding 50 µl of digest mix, and samples were incubated at 37°C for 6 h. Hydrolyzed samples were brought to 1 ml by adding HPLC-grade H_2_O.

### Calibration Standards

Calibration standards for 5′methyl- deoxycytidine ((5Me)dC) and deoxycytidine (dC) were purchased from Sigma, and dissolved in LC-MS grade water (stock solutions). A calibration series was prepared for 5(Me)dc and dC in a range of 0.1–10 ppb and 10–100 ppb respectively from the stock solutions. The same calibration standards were used in all of the experiments.

### LC-ESI-MS/MS Instrumental Analysis

Global DNA methylation was obtained by quantifying (5Me)dC and dC using ultra-pressure liquid chromatography (UPLC) for fraction separation and tandem mass spectrometry (MS-MS) for quantification. Analyses were carried out on Waters Acquity UPLC equipped with autosampler and Micromass MS Technologies Quattro Premier mass spectrometer. A 10 µl sample was introduced on an Acquity UPLC BEH C_18_, 50 mm×2.1 mm, 1.7 µm column, held at 40°C. Mobile phase used for chromatographic separation was a mixture of 0.1% formic acid in water (A) and 0.1% formic acid in acetonitrile (B) using the following gradient: 0 min: 90% A and 10% B, 2–2.5 min: 100% B, 3.9–4.0 min: 90% A and 10% B at a flow rate of 0.35 ml/min. All mobile phase constituents were LC-MS grade and were purchased from Biosolve (Valkenswaard, the Netherlands).

First, we performed full-scan spectrum under electrospray ionization (ESI) conditions. In full scan spectrum, sodium adducts 5(Me)dC/dC [M+Na]+ and 5(Me)dC-dC dimers were also observed, which is a common phenomenon in an ESI-MS full scan [23]. Analyses were performed in ESI+ mode and a multiple reaction monitoring (MRM) method was used with argon as the collision gas at a pressure of 2.88 10^−3^ mbar. Transitions monitored were m/z 242.00→125.85 for 5(Me)dC (cone voltage 14 V, collision energy 10 eV) and m/z 228.10→112.00 for dC (cone voltage 14 V, collision energy 17 eV). Dwell time per transition was 100 ms.

### Calibration Curve

We observed linear response of standards over a range of concentrations (0.1–10 ppb and 10–100 ppb) for 5(Me)dC and dC with correlation coefficients of 0.9991 and 0.9970 respectively.

### Statistics

The percentage of global DNA methylation was calculated per chemical dose and is expressed as (5Me)dC/[(5Me)dC+dC] %. We used marginal model to explore factors accounted for in the observed variation in global DNA methylation in TK6 cells, i.e., chemicals, dose and S9. Residuals were plotted to verify the assumptions of normality in the marginal model. The Shapiro-Wilk test for residuals was shown to be non-significant, which implied that approximating a response to a normal distribution was appropriate. The SAS 9.2 statistical package was used to fit the marginal model. Box plots were generated for chemicals with a significant effect on global DNA methylation in TK6 cells using SPSS v.18.

## Results

**Figure 1:**
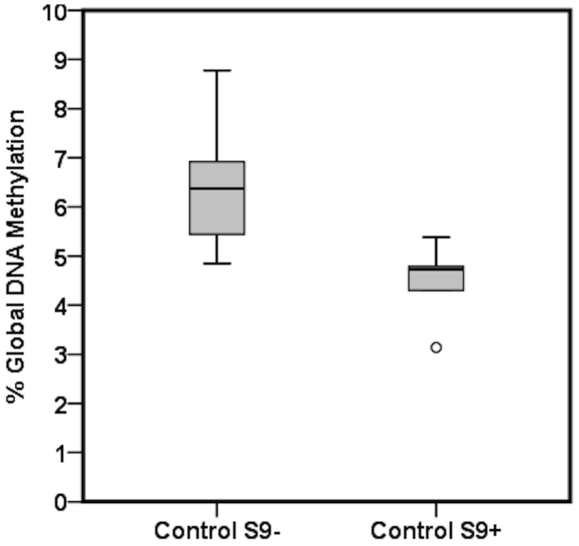
Global DNA methylation in TK6 cells cultured without S9 (control S9−) and with S9 (control S9+) is shown in the box plot. Global DNA methylation is expressed as a percentage of 5-methylcytosine versus the total number of cytosines present in the genome. The box plot describes the median (line across the box), inter-quartile range and maximum and minimum values (whiskers). Outliers are shown as open circles outside the ends of whiskers.

Global DNA methylation in control and exposed cultures per chemical dose without and with S9 metabolic mix is given in Table 1 and 2 respectively. Our results show induction of global DNA hypomethylation in response to S9 metabolic mix as shown in Figure 1.

**Table 1:** Global DNA methylation in TK6 cells per chemical dose in the absence of S9 metabolic mix.

Chemicals exposed to TK6 cells *in vitro*	Global DNA Methylation in TK6 Cells (S9−)		
	mean, +/− SD		
	Low Dose	Medium Dose	High Dose
**Control S9−**	6.38, +/−1.21		
**Formaldehyde**	4.09, +/−0.23	5.21, +/−0.57	4.61, +/−0.23
**Styrene**	4.67*	3.91, +/−0.09	4.42*
**Styrene oxide**	6.41*	6.05, +/−0.64	4.95, +/−0.39
**Benzene**	4.71, +/−0.06	4.31, +/−0.61	4.51, +/−0.03
**Hydroquinone**	3.71, +/−0.21	3.51, +/−0.42	5.31, +/−0.57
**Mitomycin C**	7.23*	4.52, +/−0.12	6.35, +/−0.63
**Ethylenedibromide**	**	3.41, +/−0.37	3.29, +/−0.43
**Epichlorohydrin**	3.81, +/−0.72	4.44, +/−0.57	4.42, +/−0.62
**Acrylamide**	5.12, +/−0.08	3.21, +/−0.1	4.72, +/−0.33
**Trichloroethylene**	**	5.32, +/−0.13	5.91, +/−0.3
**Carbon tetrachloride**	4.61, +/−0.55	4.21, +/−0.07	4.31, +/−0.02
**Cyclophosphamide**	**	4.12, +/−0.43	8.24*
**Benzo[a]fluoranthene**	4.11, +/−0.3	5.84, +/−1.3	**
**Benzo[a]pyrene**	7.51, +/−1.47	4.36, +/−0.22	7.43, +/−0.94
**Benz[a]anthracene**	6.55*	3.74, +/−0.08	6.09*

Global DNA methylation is expressed as a percentage of 5-methylcytosine versus the total number of cytosines present in the genome.

SD: Standard deviation,

* standard deviation could not be calculated because sample replicates did not pass the quality control,

** global DNA methylation values are not calculated because samples did not pass the quality control.

**Table 2:** Global DNA methylation in TK6 cells per chemical dose in the presence of S9 metabolic mix.

Chemicals exposed to TK6 cells *in vitro*	Global DNA Methylation in TK6 Cells (S9+)		
	mean, +/− SD		
	Low Dose	Medium Dose	High Dose
**Control S9+**	4.46, +/−0.83		
**Formaldehyde**	4.61, +/−0.44	4.55, +/−0.43	4.23*
**Styrene**	4.49, +/−0.19	3.13, +/−2.35	1.67*
**Styrene oxide**	5.11*	5.03, +/−0.72	3.33*
**Benzene**	**	2.92*	3.99, +/−0.05
**Hydroquinone**	**	4.36, +/−0.37	1.77*
**Mitomycin C**	5.16, +/−0.51	5.17, +/−0.31	6.22, +/−0.51
**Ethylenedibromide**	5.24, +/−1.27	4.53, +/−0.06	4.09, +/−0.29
**Epichlorohydrin**	3.89, +/−0.51	4.62, +/−0.62	3.85, +/−0.14
**Acrylamide**	3.71*	4.41, +/−0.19	3.95, +/−0.24
**Trichloroethylene**	3.61, +/−2.65	1.72*	2.59, +/−0.74
**Carbon tetrachloride**	**	3.86, +/−0.97	3.72*
**Cyclophosphamide**	**	2.85, +/−1.81	4.93*
**Benzo[a]fluoranthene**	4.36, +/−0.04	3.38, +/−0.16	4.28, +/−0.65
**Benzo[a]pyrene**	6.45*	4.85, +/−0.27	5.28, +/−0.07
**Benz[a]anthracene**	4.16, +/−0.75	4.95, +/−0.66	4.62*

Global DNA methylation is expressed as a percentage of 5-methylcytosine versus the total number of cytosines present in the genome.

SD: Standard deviation,

* standard deviation could not be calculated because sample replicates did not pass the quality control,

** global DNA methylation values are not calculated because samples did not pass the quality control.

Variation in global DNA methylation of control and exposed cultures demonstrated normal distribution (Figure S1). Assuming global DNA methylation to be normally distributed, and considering each chemical exposure to be independent but replication within exposure to be correlated, a marginal model, which captures this dependency, was applied. Covariance between model residuals, which corresponds to uniform correlation within repeated samples, was estimated to be 0.54. Ignoring the correlation within replicated exposures could result in an in accurate estimate of the significance of global DNA methylation. In our results, we observed chemicals and S9 accounting for the observed variability in global DNA methylation in TK6 cells (Table S2). Dose was found to be non-significant even in the absence of S9 in the marginal model. The model was refitted excluding the dose and the results are given in Table 3.

**Table 3:** The effect of S9 metabolic mix and carcinogens on global DNA methylation in TK6 cells *in vitro*.

Effect	Estimate	Standard Error	*t*-Value	*p*-Value|
**S9**	−0.9082	0.1956	−4.64	<. 0001*
**Formaldehyde**	−0.9032-	0.6676	−1.35	0.1806
**Styrene**	−1.7332	0.6676	−2.60	0.0115*
**Styrene oxide**	−0.2999	0.6676	−0.45	0.6547
**Benzene**	−1.5289	0.6877	−2.22	0.0295*
**Hydroquinone**	−1.8029	0.6877	−2.62	0.0108*
**Mitomycin C**	0.3268	0.6676	0.49	0.6261
**Ethylenedibromide**	−0.9566	0.6676	−1.43	0.1565
**Epichlorohydrin**	−1.2766	0.6676	−1.91	0.0601
**Acrylamide**	−1.2649	0.6676	−1.89	0.0624
**Trichloroethylene**	−1.5302	0.6879	−2.22	0.0294*
**Carbon tetrachloride**	−1.3879	0.6877	−2.02	0.0475*
**Cyclophosphamide**	−0.4141	0.7163	−0.58	0.5651
**Benzo[a]fluoranthene**	−0.9712	0.6879	−1.41	0.1626
**Benzo[a]pyrene**	0.5434	0.6676	0.81	0.4185
**Benz[a]anthracene**	−0.4332	0.6676	−0.65	0.5186

The table gives parameter estimates and standard errors for a random intercept model with chemicals and S9 as fixed effects.

* Significant at α level of 0.05.

**Figure 2:**
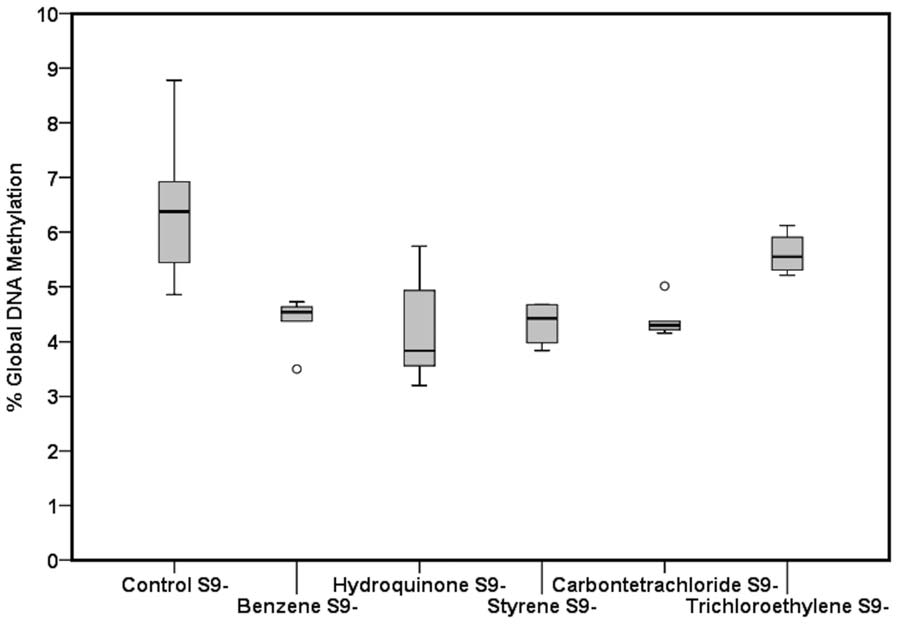
Box plot representation of global DNA methylation in control TK6 cells and TK6 cells exposed with benzene, hydroquinone, styrene, carbon tetrachloride and trichloroethylene without S9 metabolic mix. Global DNA methylation is expressed as percentage of 5-methylcytosine versus the total number of cytosines present in the genome. The box plot describes the median (line across the box), inter-quartile range and maximum and minimum values (whiskers). Outliers are shown as open circles outside the ends of whiskers.

**Figure 3:**
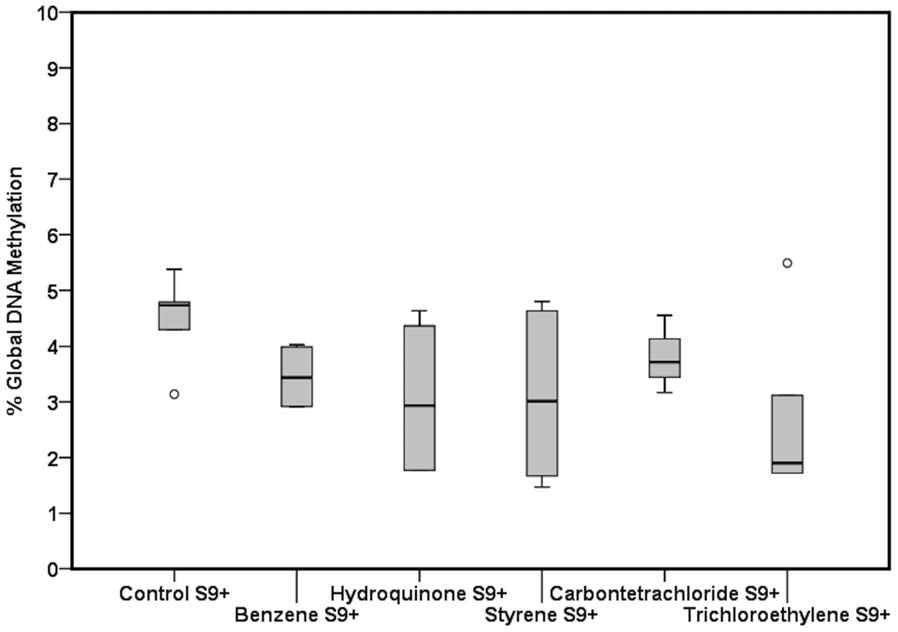
Box plot representation of global DNA methylation in control TK6 cells and TK6 cells exposed with benzene, hydroquinone, styrene, carbon tetrachloride and trichloroethylene with S9 metabolic mix. Global DNA methylation is expressed as percentage of 5-methylcytosine versus the total number of cytosines present in the genome. The box plot describes the median (line across the box), inter-quartile range and maximum and minimum values (whiskers). Outliers are shown as open circles outside the ends of whiskers.

Furthermore, we show that benzene and its metabolite hydroquinone, and styrene, carbon tetrachloride and trichloroethylene significantly affected the global DNA methylation in TK6 cells (Table 3). Global DNA methylation profiles observed with exposure to these chemicals in TK6 cells without and with S9 are shown in Figure 2 and 3 respectively.

## Discussion

The classical theory of carcinogenesis is driven by genetic mutations and chromosomal abnormalities conferring genome instability [24], [25]. However, the current study highlights the importance of global DNA methylation as an early epigenetic factor in response to genotoxic exposure.

Indirect- acting carcinogens require metabolic activation to become reactive carcinogens. Due to the required metabolic activation, a mixture of S9 liver extract (1% v/v) was added to half of the cultures. S9 mixture contains enzymes required for phase-I metabolic activation of xenobiotics. Expression of metabolic enzymes is linked to reactive oxidative stress pathways [26]. Oxidative stress affects DNA methylation by altering the S-adenosylmethionine (SAM) and S-adenosylhomocysteine (SAH) ratio [27]–[29]. In this study, the addition of S9 metabolic mix in TK6 cell cultures resulted in global DNA hypomethylation (β = −0.9082, *p*<0.0001) (Table 3, Figure 1). S9-induced global DNA hypomethylation in these cultures could be mechanistically linked to the induction of oxidative stress pathways. Oxidative stress activates cellular and nuclear signaling pathways, which have intrinsic histone acetyltransferase (HAT) and histone deacetylase (HADC) activities. In turn, these proteins are linked to DNA methyltransferases (DNMTs) in the nuclear pathways leading to the conformational changes in histones and chromatin structure, and thus they alter the cellular transcription level [30], [31].

A number of chemicals used in this study affected global DNA methylation changes in TK6 cells (Table 3, Figure 2 and 3). We observed interesting global DNA methylation patterns. Benzene (β = −1.5289, *p*<0.0295) and it metabolite hydroquinone (β = −1.8029, *p*<0.0108) exposure induced global DNA hypomethylation in TK6 cells, while styrene exposure (β = −1.7332, *p*<0.0115) induced global DNA hypomethylation but its metabolite styrene oxide exposure did not affect the global DNA methylation in TK6 cells (β = −0.2999, *p*<0.6547). Benzene exposure has shown to be linked to reduced methylation levels of DNA repetitive elements [32]. Benzene and hydroquinone exposure activates the oxidative stress pathways in cells which affects the cellular DNA methylation pattern [33]. Styrene exposure induces DNA adduct formation and oxidative stress in cells [34]. Besides these effects, we report the induction of global DNA hypomethylation by styrene as a potential non-genotoxic mechanism, which could account for its toxicity. We also exposed TK6 cells to carbon tetrachloride and trichloroethylene. These chemicals mainly act through the formation of reactive intermediates after the metabolic activation. In the current study, we observed global DNA hypomethylation induced by carbon tetrachloride (β = −1.3879, *p*<0.0475) and trichloroethylene (β = −1.5302, *p*<0.0294) exposure in TK6 cells (Table 3, Figure 2 and 3). Previous studies also reported similar findings about carbon tetrachloride and trichloroethylene (TCE) induced global DNA hypomethylation. Carbon tetrachloride induced global DNA hypomethylation was rescued by supplementation with S-adenosylmethionine (SAM) in rat liver [35], [36]. These observations suggested that carbon tetrachloride induced DNA hypomethylation involved methionine metabolic pathways. In addition, these chemicals induce oxidative stress, which could affect the cellular methylome.

In contrast to other studies, we did not observe global DNA methylation changes in TK6 cells by exposure to poly-aromatic hydrocarbons (PAHs). Chronic exposure of benzo[a]pyrene to mouse embryonic fibroblasts *in vitro* induced global DNA hypermethylation [37]. Also, differences in DNA methylation levels have been reported in peripheral blood lymphocytes of workers chronically exposed to PAH compared to their matched controls [38]. Different experimental settings used in these studies compared to the current study could explain the heterogeneity observed in PAHs induced DNA methylation changes. Furthermore, no global DNA methylation changes in TK6 cells were observed for mitomycin C, formalin, cyclophosphamide, ethylenedibromide, epichlorohydrin and acrylamide. Global DNA methylation changes in response to these chemicals have not been reported elsewhere. Subtle epigenetic effects, such as histone modifications and gene specific DNA methylation, in response to these chemicals could not be ignored and will be explored further.

Global DNA hypomethylation in TK6 cells induced by direct and indirect- acting genotoxic carcinogens investigated in this study could imply that cells are under pre-neoplastic conditions. If sustained global DNA hypomethylation persists, this could drive these cells to neoplastic phenotype. However, the duration and extent of exposure required for sustained global DNA hypomethylation to confer neoplastic phenotype needs to be fully understood.

### Conclusion

In conclusion, we report the non-genotoxic effect, i.e., alteration in global DNA methylation, in response to a number of carcinogens, which are traditionally considered to act through genotoxic mechanisms. We also describe that S9 metabolic mix alters the global DNA methylation pattern in TK6 cells. Future work will address the dose-dependent effects of S9 metabolic mix *in vitro* and the pathways involved in carcinogen-induced DNA methylation changes. Our results suggest the use of different cell lines and more varied assays to validate the above findings, and to explore the mechanistic links.

## Supporting Information

Figure S1 **Histogram and density plot of residuals to assess normality.** Normality assumption of response (global DNA methylation) was assessed by plotting the residuals (x-axis). The plot appears to indicate that this assumption is plausible. Shapiro-Wilk test was also performed to confirm normality and residuals were shown to be non-significant.
(TIF)

Table S1 **Overview of agents, their classification and administered doses used in the treatment of TK6 cells.**
(DOCX)

Table S2 **Results of the marginal model describing the effect of exposure, i.e., chemicals, dose, and S9, on global DNA methylation in TK6 cells** ***in vitro*** **.**
(DOCX)
