# Supplementary figures and images for: Epigenetic Factors in Cancer Risk: Effect of Chemical Carcinogens on Global DNA Methylation Pattern in Human TK6 Cells
Source: PLoS One. 2012 Apr 11;7(4):e34674. doi: 10.1371/journal.pone.0034674 (PMC3324488; doi:10.1371/journal.pone.0034674)

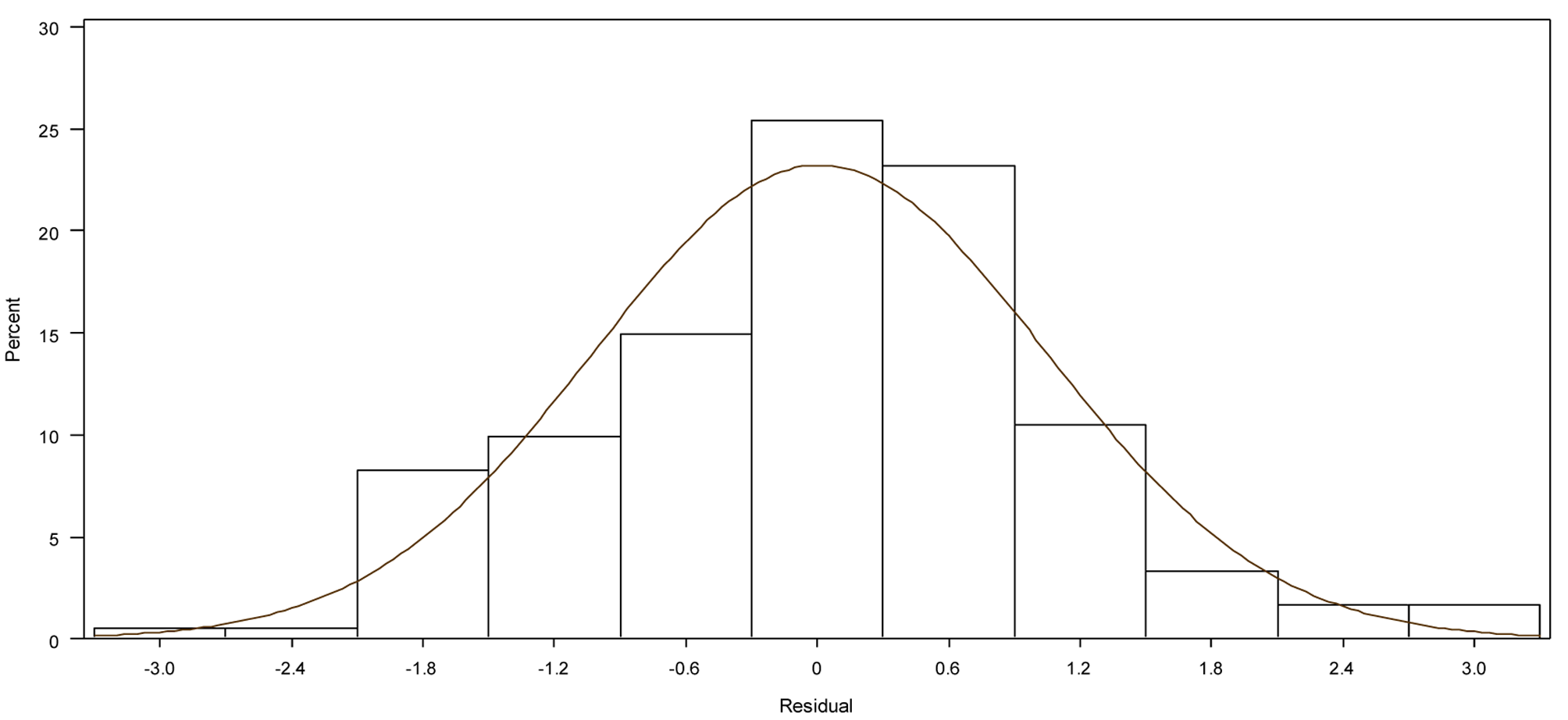

Supplement: Figure S1 — Histogram and density plot of residuals to assess normality. Normality assumption of response (global DNA methylation) was assessed by plotting the residuals (x-axis). The plot appears to indicate that this assumption is plausible. Shapiro-Wilk test was also performed to confirm normality and residuals were shown to be non-significant. (TIF) [file pone.0034674.s001.tif]
