# Supplementary material for: Epigenetic Factors in Cancer Risk: Effect of Chemical Carcinogens on Global DNA Methylation Pattern in Human TK6 Cells
Source: PLoS One. 2012 Apr 11;7(4):e34674. doi: 10.1371/journal.pone.0034674 (PMC3324488; doi:10.1371/journal.pone.0034674)
Supplement: Table S1 — Overview of agents, their classification and administered doses used in the treatment of TK6 cells. (DOCX) [file pone.0034674.s002.docx]

Table S1

| Agents | IARC^§^ | Category | Concentration (µM) | | |
| --- | --- | --- | --- | --- | --- |
|  |  |  | High | Medium | Low |
| Formaldehyde^*,1,2^ | 1 | Aldehyde | 100 | 10 | 1 |
| Styrene^**,1^ | 2B | Aromatic hydrocarbon | 5000 | 500 | 50 |
| Styrene 7,8-oxide^*,1^ | 2A | Aromatic hydrocarbon | 500 | 50 | 5 |
| Benzene^**,1^ | 1 | Aromatic hydrocarbon | 100 | 10 | 1 |
| Hydroquinone^*,1^ | 3 | Aromatic hydrocarbon | 0.5 | 0.05 | 0.005 |
| Mitomycin C^*,2^ | 2B | Cytostaticum | 0.5 | 0.05 | 0.005 |
| Ethylenedibromide^**,1,2^ | 2A | Organobromide | 1000 | 100 | 10 |
| Epichlorohydrin^*,1^ | 2A | Organochloride | 500 | 50 | 5 |
| Acrylamide^**,1^ | 2A | Amide | 500 | 50 | 5 |
| Trichloroethylene^**,1^ | 2A | Chlorinated hydrocarbon | 5000 | 500 | 50 |
| Carbontetrachloride^**,1^ | 2B | Chlorinated hydrocarbon | 1000 | 100 | 10 |
| Cyclophosphamide^**,1^ | 1 | Cytostaticum | 50 | 5 | 0.5 |
| Benzo[a]fluoranthene^**,1^ | 2B | Poly aromatic hydrocarbon | 500 | 50 | 5 |
| Benzo[a]pyrene^**,1^ | 1 | Poly aromatic hydrocarbon | 500 | 50 | 5 |
| Benz[a]anthracene^**,1^ | 2B | Poly aromatic hydrocarbon | 500 | 50 | 5 |

^§^International agency for research on cancer; *Direct acting agent; **Indirect acting agent, 1: DNA adduct forming agent; 2: DNA Cross linking agent
