# Supplementary material for: Epigenetic Factors in Cancer Risk: Effect of Chemical Carcinogens on Global DNA Methylation Pattern in Human TK6 Cells
Source: PLoS One. 2012 Apr 11;7(4):e34674. doi: 10.1371/journal.pone.0034674 (PMC3324488; doi:10.1371/journal.pone.0034674)
Supplement: Table S2 — Results of the marginal model describing the effect of exposure, i.e., chemicals, dose, and S9, on global DNA methylation in TK6 cells in vitro . (DOCX) [file pone.0034674.s003.docx]

Table S2

| **Effect** | **Num DF**** | **Den DF***** | **F Value** | ***p-*Value** |
| --- | --- | --- | --- | --- |
| **Chemicals** | 15 | 67 | 3.72 | <.0001^*^ |
| **S9** | 1 | 67 | 21.41 | <.0001^*^ |
| **Dose** | 1 | 67 | 2.74 | 0.1024 |

^*^Significant at α level of 0.05, ******Num DF: Numerator Degree of Freedom, *******Den DF: Denominator Degree of Freedom
